# Disorder-specific and shared neurophysiological impairments of attention and inhibition in women with attention-deficit/hyperactivity disorder and women with bipolar disorder

**DOI:** 10.1017/S0033291715001877

**Published:** 2015-11-10

**Authors:** G. Michelini, G. L. Kitsune, G. M. Hosang, P. Asherson, G. McLoughlin, J. Kuntsi

**Affiliations:** 1 King's College London, MRC Social, Genetic and Developmental Psychiatry Centre, Institute of Psychiatry, Psychology and Neuroscience, London, UK; 2 Department of Psychological Medicine, King's College London, Institute of Psychiatry, Psychology and Neuroscience, London, UK; 3 Department of Psychology, Goldsmiths, University of London, London, UK

**Keywords:** Attention, attention-deficit/hyperactivity disorder, bipolar disorder, conflict monitoring, event-related potentials, inhibitory control

## Abstract

**Background:**

In adults, attention-deficit/hyperactivity disorder (ADHD) and bipolar disorder (BD) have certain overlapping symptoms, which can lead to uncertainty regarding the boundaries of the two disorders. Despite evidence of cognitive impairments in both disorders separately, such as in attentional and inhibitory processes, data on direct comparisons across ADHD and BD on cognitive–neurophysiological measures are as yet limited.

**Method:**

We directly compared cognitive performance and event-related potential measures from a cued continuous performance test in 20 women with ADHD, 20 women with BD (currently euthymic) and 20 control women.

**Results:**

The NoGo-N2 was attenuated in women with BD, reflecting reduced conflict monitoring, compared with women with ADHD and controls (both *p* < 0.05). Both ADHD and BD groups showed a reduced NoGo-P3, reflecting inhibitory control, compared with controls (both *p* < 0.05). In addition, the contingent negative variation was significantly reduced in the ADHD group (*p* = 0.05), with a trend in the BD group (*p* = 0.07), compared with controls.

**Conclusions:**

These findings indicate potential disorder-specific (conflict monitoring) and overlapping (inhibitory control, and potentially response preparation) neurophysiological impairments in women with ADHD and women with BD. The identified neurophysiological parameters further our understanding of neurophysiological impairments in women with ADHD and BD, and are candidate biomarkers that may aid in the identification of the diagnostic boundaries of the two disorders.

## Introduction

Attention-deficit/hyperactivity disorder (ADHD) and bipolar disorder (BD) are common psychiatric conditions in adults, affecting around 2–4% and 1–2% of the adult population, respectively (Merikangas *et al.* 2011; Willcutt, 2012). Although ADHD and BD represent distinct conditions, their diagnostic formulations present certain areas of symptomatic overlap. In adults, ADHD may be manifest with some symptoms common to mania/hypomania, such as distractibility, psychomotor restlessness and talkativeness (Skirrow *et al.* 2012; Asherson *et al.* 2014). Additionally, both disorders are associated with features of mood dysregulation, such as irritability and emotional lability (Skirrow *et al.* 2012, 2014; GL Kitsune *et al.* unpublished observations). Of note, ADHD symptoms are chronic and trait-like, while BD symptoms of mania and depression tend to occur for a distinct period of time (Asherson *et al.* 2014). Yet, individuals with BD may still show residual symptoms of distractibility and mood dysregulation (overlapping with ADHD), and residual cognitive and functional impairments between episodes (Torres *et al.* 2007; Henry *et al.* 2013). Importantly, symptomatic similarities can result in uncertainty regarding the boundaries of the two disorders, and difficulties in distinguishing between the two disorders in some patients, which in turn may result in inappropriate treatment decisions (Asherson *et al.* 2014).

Adults with ADHD or BD may display similar cognitive impairments. For example, both ADHD and euthymic BD are associated with poor accuracy in attentional and inhibitory processing tasks (Robinson *et al.* 2006; McLoughlin *et al.* 2010; Torralva *et al.* 2011) and increased reaction time variability (RTV), which may reflect short-term fluctuations in attentional performance (Brotman *et al.* 2009; Kuntsi *et al.* 2010; Kuntsi & Klein, 2012). Comparative studies across ADHD and BD, using identical measures, may aid in the identification of attentional and inhibitory deficits underlying overlapping symptoms and functional impairment, yet empirical data are currently limited.

The investigation of neurophysiological processes with event-related potentials (ERPs) provides a direct measure of covert brain activity underlying behavioural performance with millisecond temporal resolution, and may enable a sensitive comparison of cognitive profiles in ADHD and BD (Banaschewski & Brandeis, 2007; McLoughlin *et al.* 2014*a*). Several previous studies on attentional and inhibitory processing in ADHD have explored ERPs during the cued continuous performance test (CPT-OX), which involves presentation of cue, target (Go) and non-target (NoGo) stimuli and requires a response only when a target follows a cue (van Leeuwen *et al.* 1998; Banaschewski *et al.* 2004). A reduced fronto-central P3 has consistently been reported in response to NoGo stimuli (NoGo-P3) in children, adolescents and adults with ADHD compared with controls, reflecting abnormal inhibitory control (Valko *et al.* 2009; Doehnert *et al.* 2010; McLoughlin *et al.* 2010, 2011; Albrecht *et al.* 2013; Tye *et al.* 2014). Attenuations in a parietal P3 after presentation of cue stimuli (Cue-P3) and in the subsequent contingent negative variation (CNV), a late negative potential before the occurrence of the next stimulus, have also been found in individuals with ADHD, reflecting impaired attentional orienting and response preparation, respectively (Doehnert *et al.* 2010; McLoughlin *et al.* 2010, 2011; Albrecht *et al.* 2013), although case–control differences in these components have not been reported in all studies (Dhar *et al.* 2010; Skirrow, 2012). Differences between adults with ADHD and control adults are generally not found in other ERP components elicited by this task; such as the P3 in response to target (Go-P3), reflecting response execution, and the N2 to non-target stimuli (NoGo-N2), indexing conflict monitoring, which refers to the ability to monitor ongoing behaviour, detect conflict and adjust response selection (Yeung & Cohen, 2006; McLoughlin *et al.* 2010). N2 deflections are particularly elicited by high-conflict trials, such as non-target or incongruent stimuli, and are attenuated in ADHD individuals in paradigms inducing higher conflict-monitoring demands than the CPT-OX, such as flanker tasks, suggesting possible modulations of this component by task and stimuli (Barry *et al.* 2009; McLoughlin *et al.* 2009, 2014*b*).

In ERP studies, BD has been associated with attenuations in early sensory and attentional ERP components (e.g. mismatch negativity, P50 and P2) in auditory tasks (Hall *et al.* 2007; Jahshan *et al.* 2012; Cabranes *et al.* 2013; Swann *et al.* 2013). Reduced P3 enhancements to target stimuli have been reported in adults with BD in studies using a visual paradigm with standard, deviant and target conditions (Maekawa *et al.* 2013) and using an oddball paradigm (Hall *et al.* 2007), but not in all studies (Schulze *et al.* 2008; Bestelmeyer, 2012). Some evidence also indicates impairments in conflict monitoring in adults with BD, indexed by reduced N2 in response to target stimuli with an auditory oddball task (Ethridge *et al.* 2012) and reduced error-related negativity (ERN) in error responses (Morsel *et al.* 2014). Despite initial evidence that may suggest impairments in ERPs of attentional and inhibitory processing in BD, however, ERP data on these processes are limited, and no studies, to our knowledge, have used the CPT-OX.

Direct comparisons on cognitive performance and ERP measures in ADHD and BD are sparse. One study on adults with ADHD and adults with BD investigating ERP measures of reward processing found significant differences in the amplitude of a reward-sensitive P3, which was attenuated in ADHD but enhanced in BD participants compared with controls (Ibanez *et al.* 2012). However, no study to date has compared ERP components associated with attentional and inhibitory processing in both disorders using the CPT-OX. In addition, most studies of this kind, especially on ADHD, have used male samples because, among children, ADHD is more prevalent in males than in females, and very little is known about these processes in females. Yet, a similar prevalence of ADHD has been reported in both adult men and women (Faraone & Biederman, 2005; Das *et al.* 2012). Similarly, comparable gender ratios have been found for BD in adults (Pini *et al.* 2005).

The aim of the current study was to directly compare cognitive performance and ERP measures associated with attentional and inhibitory processing in ADHD and BD in adults. This study was conducted on an all-female sample, in order to match the groups on gender but also to explore the neglected area of ERP indices associated with these processes in females. Based on previous studies of male participants (McLoughlin *et al.* 2010; Albrecht *et al.* 2013; Doehnert *et al.* 2013), we predicted that women with ADHD would show reduced NoGo-P3, Cue-P3 and CNV, but normal NoGo-N2. Given the limited and mixed results in ERP studies of BD individuals and the lack of similar studies using the CPT-OX, we adopted an exploratory approach for the BD group and for the comparison with ADHD.

## Method

### Sample

The sample for this study consisted of 60 adult women aged between 20 and 52 years, divided into three groups: 20 with ADHD, 20 with BD and 20 controls. Participants with ADHD were recruited from the National Adult ADHD Clinic at the Maudsley Hospital, where any female cases meeting inclusion criteria were considered for potential inclusion in the study. Participants with BD were recruited from the Maudsley Psychosis Clinic and a sample that had previously participated in another research study (Hosang *et al.* 2012). Control participants were recruited from the Mindsearch volunteer database maintained by the Institute of Psychiatry, King's College London, which comprises several thousand potential participants. Participants were randomly selected from all those meeting recruitment criteria for this study.

Diagnosis in the clinical groups was confirmed by checking medical records for details of diagnosis and psychiatric history, following Diagnostic and Statistical Manual of Mental Disorders, 4th edition (DSM-IV) criteria (APA, 2000). All of the ADHD participants had a current combined-type diagnosis or a current inattentive-type diagnosis with sufficient symptoms of hyperactivity–impulsivity in childhood to meet a childhood combined-type diagnosis. Participants in the BD group had a diagnosis of BD type I, having experienced at least one manic episode in the past. Those who were experiencing a manic episode at the time of the assessment were excluded; all participants included in the BD group were currently euthymic. Exclusion criteria for all groups were drug or alcohol dependency in the last 6 months, autism, epilepsy, neurological disorders, brain injury, past electroconvulsive therapy, current involvement in another research trial likely to alter symptom severity, pregnancy or a limited proficiency in the English language. Individuals with ADHD and individuals with BD with a reported co-morbidity of both ADHD and BD were also excluded. Control participants, who reported a history of psychiatric disorders or who were taking psychiatric medication, were excluded from the study. Co-morbidity in the clinical groups and lack of psychiatric disorders in the control group were further assessed through clinical evaluations when participants underwent the cognitive–electroencephalographic (EEG) assessment for this study. Further details on the clinical assessment of this sample can be found elsewhere (GL Kitsune *et al.* unpublished observations). In brief, ADHD was excluded in the BD group after conducting the Diagnostic Interview for ADHD in Adults (DIVA v. 2.0; Kooij & Francken, 2007). BD was excluded in the ADHD group by checking for a history of past episodes of depression or hypomania/mania and evaluating current mood symptoms using the Altman Self-Rating Mania Scale (Altman *et al.* 1997) and the Beck Depression Inventory (Beck *et al.* 1996), and current and lifetime ever symptoms using the Young Mania Rating Scale (Young *et al.* 1978). The ADHD and BD groups did not differ significantly on any of the mood scales for current symptoms (GL Kitsune *et al.* unpublished observations).

All participants had normal or corrected-to-normal vision. Mean age did not differ by group (*F*_2,59_ = 1.63, *p* = 0.21), with a mean age of 37.40 (s.d. = 7.70) for the ADHD group, 40.30 (s.d. = 7.70) for the BD group and 36.7 (s.d. = 4.30) for the control group. Participants’ intelligence quotients (IQs) were assessed with the Wechsler Abbreviated Scale of Intelligence, fourth edition (Wechsler, 1999) and did not differ between groups (*F*_2,58_ = 1.37, *p* = 0.26), with mean IQs of 104 (s.d. = 17.90) for ADHD, 108 (s.d. = 12.50) for BD and 112 (s.d. = 14.20) for control participants. Participants with ADHD were asked to stop taking any stimulant medication prescribed for their ADHD 48 h prior to the assessment. For ethical reasons, participants were not asked to stop taking mood stabilizers (70% of the BD group), anti-psychotic medication (40% of the BD group) or anti-depressants (7% of the ADHD group and 25% of the BD group) they had been prescribed. All participants were asked to refrain from caffeinated drinks and nicotine 2 h prior to the testing session. Ethical approval for the study was granted by the Camberwell St Giles Research Ethics Committee (approval number 11/LO/0438) and all participants provided informed consent.

### Procedure and cognitive performance measures

Participants attended a single 4.5 h research session (including breaks) for cognitive–EEG assessment, IQ assessment and clinical interviews. The task was a CPT-OX, flanker version (Doehnert *et al.* 2008; McLoughlin *et al.* 2010, 2011). This is a cued-Go/NoGo task that probes attention, preparation and response inhibition or control. The task consists of 400 letter arrays formed of a centre letter with incompatible flankers on each side to increase difficulty for adults. Each letter array was presented for 150 ms with a stimulus onset asynchrony (SOA) of 1.65 s in a pseudo-randomized order at the centre of a computer monitor. The tasks involved the presentation of 80 cues (XOX) followed either by 40 Go (OXO) and 40 NoGo (XDX) stimuli, alternated with random letter sequences as distractors. Participants were instructed to respond only to Cue-Go sequences by pressing a button as quickly as possible with the digit finger of their preferred hand, and to withhold the response in presence of a NoGo stimulus, of a Go not preceded by a cue (40 trials), or of any other irrelevant letters. The task was practised prior to task performance and lasted 11 min. The task followed a 2 × 3 min resting-state recording, and was run as first in a battery of three cognitive–EEG tasks.

Cognitive performance measures included target mean reaction time (MRT, i.e. mean latency of responding in ms after target onset), RTV (measured as standard deviation of target reaction time) and number of errors. MRT and RTV were calculated across correctly answered Go trials. Errors included omission errors (non-response to Go trials), total commission errors (response to cue, NoGo or distractor stimuli) and OXO-not-XOX commission errors (response to a Go not following a cue).

### Electrophysiological recording and analysis

The EEG was recorded from a 62-channel DC-coupled recording system (extended 10–20 montage), using a 500 Hz sampling rate, impedances under 10 kΩ, and FCz as the recording reference. The electro-oculograms were recorded from electrodes above and below the left eye and at the outer canthi. The EEG data were analysed using Brain Vision Analyser 2.0 (Brain Products). Researchers were blind to group status during EEG pre-processing and analysis. Raw EEG recordings were down-sampled to 256 Hz, re-referenced to the average of all electrodes, and digitally filtered using Butterworth band-pass filters (0.1–30 Hz, 24 dB/octave). All trials were also visually inspected for electrical artefacts (due to electrical noise in the EEG recording) or obvious movement, and sections of data containing artefacts were removed manually. Ocular artefacts, corresponding to blink-related and vertical and horizontal eye movements, were identified using the infomax Independent Component Analysis algorithm (ICA; Jung *et al.* 2000), which allows for removal of the components associated with ocular artefacts by back-projection of all but those components. Sections of data with remaining artefacts exceeding ±100 *µ*V in any channel or with a voltage step greater than 50 *µ*V were automatically rejected. Baseline correction was performed using a 500-ms pre-stimulus reference period^1^†.

**Fig. 1.**
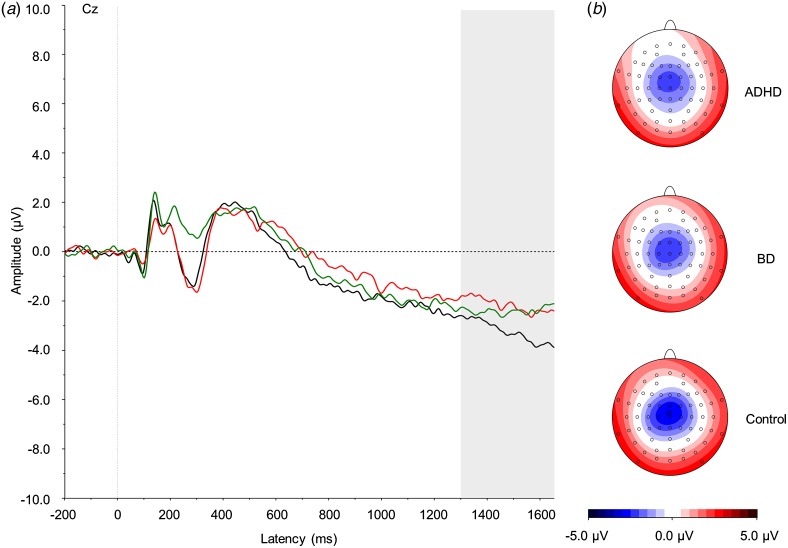
(*a*) Grand average event-related potentials to cue stimuli at the Cz electrode, showing the contingent negative variation in the 1300–1650 ms window. ADHD, Attention-deficit/hyperactivity disorder (light grey; red online); BD, bipolar disorder (mid grey; green online). Controls are shown in black. (*b*) Topographic maps for each group. For a colour figure, see the online version.

**Fig. 2.**
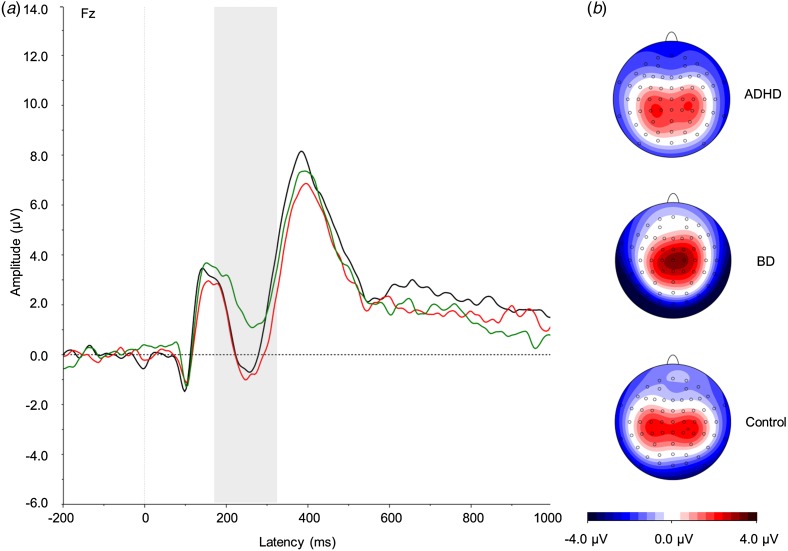
(*a*) Grand average event-related potentials to NoGo stimuli at the Fz electrode, showing the NoGo-N2 in the 175–325 ms window. ADHD, Attention-deficit/hyperactivity disorder (light grey; red online); BD, bipolar disorder (mid grey; green online). Controls are shown in black. (*b*) Topographic maps for each group. For a colour figure, see the online version.

**Fig. 3.**
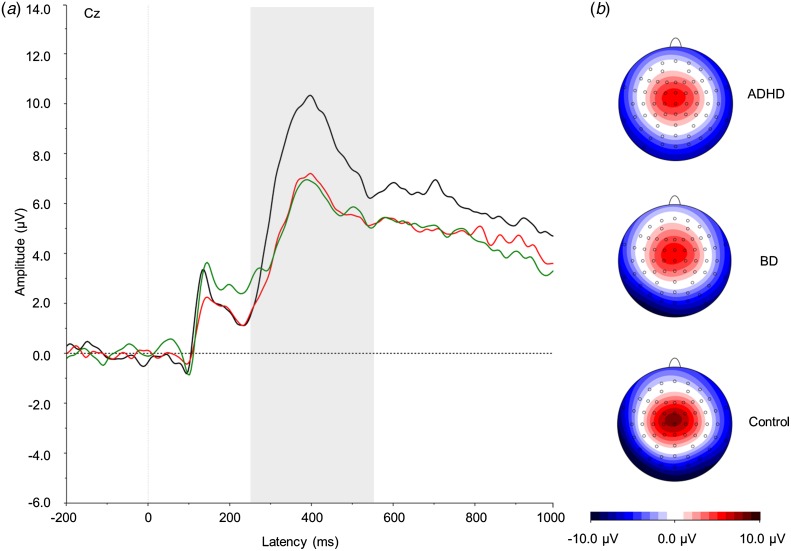
(*a*) Grand average event-related potentials to NoGo stimuli at the Cz electrode, showing the NoGo-P3 in the 250–550 ms window. ADHD, Attention-deficit/hyperactivity disorder (light grey; red online); BD, bipolar disorder (mid grey; green online). Controls are shown in black. (*b*) Topographic maps for each group. For a colour figure, see the online version.

Stimulus-locked epochs (stimulus window from −200 to 1650 ms) were averaged based on three different response conditions: cue, Go and NoGo. Averages only included trials with correct responses (Go) or correctly rejected trials (NoGo and cue) and contained at least 20 artefact-free segments (see online Supplementary material for number of segments included in the ERP average by group). ERP measures were identified within the selected electrodes and latency windows for which effects were expected to be largest, based on previous studies (McLoughlin *et al.* 2010, 2011; Albrecht *et al.* 2013; Doehnert *et al.* 2013) and verified against the topographic maps and the grand averages (Figs 1–3). ERPs were measured as the mean amplitude in the designated latency window. This approach has been adopted in previous similar studies (Groom *et al.* 2010; Tye *et al.* 2014), and has the advantage of being unaffected by latency variability (Luck, 2005). In cue trials, the P3 was measured at Pz between 300 and 650 ms, and the CNV at Cz between 1300 and 1650 ms. In NoGo trials, the N2 was measured at Fz between 175 and 325 ms, and the P3 at Cz between 250 and 550 ms. In Go trials, the P3 was measured at CPz between 250 and 500 ms. A clear N2 was not observed in Go trials, in line with other studies on tasks inducing a low-conflict-monitoring demand (Bokura *et al.* 2001; Gajewski & Falkenstein, 2013) and was not included into the analysis.

### Statistical analyses

All participants were included in the analysis of cognitive performance data. Two ADHD participants were excluded from the ERP analysis of the Go condition due to having less than 20 artefact-free segments available for analysis.

Group differences on the reaction time measures were explored using univariate analyses of variance (ANOVAs), followed by *post-hoc t* tests. MRT and RTV had skewed distributions and were log-transformed with optimized minimal skew through the ‘lnskew0’ command in Stata (Stata Corp.). Performance accuracy was generally high as errors were rare, in line with previous studies on this task (McLoughlin *et al.* 2010, 2011; Albrecht *et al.* 2013; Doehnert *et al.* 2013). Since distribution of errors was thus not normal and no transformations were successful, effects of group on these variables were entered into non-parametric analysis, using Kruskal–Wallis tests, followed by *post-hoc* Mann–Whitney *U* tests.

Group effects on ERP parameters were tested with separate ANOVAs, followed by *post-hoc t* tests. All ERP measures had normal distribution. We report both *p* values (*p* < 0.05 for significance and *p* < 0.10 for a trend) and effect sizes (Cohen's *d*) for comparisons of cognitive performance and ERP measures. Effect sizes were calculated using the difference in the means, divided by the pooled standard deviation, where *d* = 0.20 constitutes a small effect, *d* = 0.50 a medium effect and *d* = 0.80 a large effect (Cohen, 1988).

### Ethical standards

All procedures contributing to this work comply with the ethical standards of the relevant national and institutional committees on human experimentation and with the Helsinki Declaration of 1975, as revised in 2008.

## Results

### Cognitive performance measures

A trend-level effect of group emerged for RTV (*F*_2,57_ = 2.67, *p* = 0.08). *Post-hoc* analyses revealed a significant difference between the BD and control groups (*p* = 0.03) and a trend-level difference between the ADHD and control groups (*p* = 0.06) on RTV, both with medium effect sizes (Table 1), but no differences between the ADHD and BD groups (*p* = 0.93). Groups did not differ on MRT (*F*_2,57_ = 1.47, *p* = 0.24).

**Table 1.** Cognitive performance and event-related potential measures from the cued continuous performance test: means, effect sizes and significance of group comparisons

	ADHD (*n* = 20)^a^: mean (s.d.)^b^	BD (*n* = 20): mean (s.d.)^b^	Controls (*n* = 20): mean (s.d.)^b^	ADHD *v*. BD, effect size: *d*	ADHD *v*. controls, effect size: *d*	BD *v*. controls, effect size: *d*
MRT, ms	425.31 (75.74)	418.30 (67.41)	391.58 (63.68)	0.05	0.49^d^	0.44
RTV, ms	109.18 (58.83)	101.73 (37.77)	76.91 (39.24)	0.02	0.60^d^†	0.68^d^*
OE	1.10 (1.55)	1.35 (2.52)	0.60 (1.57)	0.12	0.32*	0.36†
OXO-not-XOX CE	1.05 (1.88)	0.60 (2.04)	0.50 (0.89)	0.23	0.37	0.06
Total CE	7.25 (16.03)	2.40 (5.39)	0.75 (0.97)	0.41	0.57^d^*	0.43
Cue-P3 at Pz	2.30 (1.64)	1.36 (1.80)	1.83 (2.04)	0.56^d^	0.26	0.25
CNV at Cz	−2.24 (1.03)	−2.31 (1.36)	−3.31 (2.12)	0.06	0.66^d^*	0.58^d^†
NoGo-N2 at Fz	0.57 (1.88)	2.41 (2.64)	0.84 (2.07)	0.83^c^*	0.14	0.68^d^*
NoGo-P3 at Cz	5.42 (2.73)	5.56 (3.31)	7.68 (2.57)	0.05	0.88^c^*	0.73^d^*
Go-P3 at CPz	5.01 (2.76)	5.56 (3.23)	6.10 (2.18)	0.19	0.45^d^	0.20

ADHD, Attention-deficit/hyperactivity disorder; s.d., standard deviation; BD, bipolar disorder; MRT, mean reaction time; RTV, within-subject variability in reaction times; OE, omission errors; CE, commission errors; CNV, contingent negative variation.

a Only 18 participants with ADHD were included in the average of the Go condition, as two participants did not have at least 20 artefact-free segments.

b Means and s.d.s were calculated on raw data.

c Large effect size.

d Medium effect size.

* *p* < 0.05, † *p* < 0.10.

Trend-level effects emerged on the number of total commission errors (*H*_2_ = 4.96, *p* = 0.08) and omission errors (*H*_2_ = 4.74, *p* = 0.09). *Post-hoc* analyses indicated that participants with ADHD made significantly more commission (*p* = 0.03) and omission (*p* = 0.04) errors than controls, with medium and small effect sizes, respectively (Table 1). Participants with BD showed a trend-level difference on the number of omission errors (*p* = 0.07) from controls, with a small effect size, but no difference on commission errors (*p* = 0.34). The ADHD and BD groups did not differ on commission (*p* = 0.20) or omission (*p* = 0.90) errors. No effect of group emerged for OXO-not-XOX commission errors (*H*_2_ = 3.81, *p* = 0.15).

### ERP parameters

#### Cue condition

An effect of group did not emerge on the Cue-P3 (*F*_2,57_ = 1.31, *p* = 0.28).

A trend-level effect of group emerged for the CNV (*F*_2,57_ = 2.86, *p* = 0.07). *Post-hoc* comparisons showed a significant difference between the ADHD and control groups (*p* = 0.05) and a trend-level difference between the BD and control groups (*p* = 0.09), both with medium effect size (Table 1). No difference emerged between the two clinical groups (*p* = 0.85).

#### NoGo condition

There was a significant effect of group on the NoGo-N2 (*F*_2,57_ = 4.03, *p* = 0.02). *Post-hoc* analyses revealed that the BD group significantly differed from the ADHD (*p* = 0.015) and control (*p* = 0.04) groups, with large and medium effect sizes, respectively (Table 1). The ADHD and control groups did not differ from each other (*p* = 0.66).

A significant effect of group emerged on the NoGo-P3 (*F*_2,57_ = 3.86, *p* = 0.03). *Post-hoc* analyses showed that both the ADHD (*p* = 0.01) and BD (*p* = 0.03) groups significantly differed from controls, respectively, with large and medium effect sizes (Table 1), but not from each other (*p* = 0.88).

#### Go condition

No significant effect of group emerged on the Go-P3 (*F*_2,55_ = 0.73, *p* = 0.49).

## Discussion

In a direct comparison of women with ADHD, women with BD and control women on cognitive performance and ERP measures from a CPT-OX task, we report evidence for both disorder-specific (conflict monitoring) and overlapping (inhibitory control and potentially response preparation) neurophysiological impairments across the disorders. The current study represents the first cognitive–electrophysiological investigation comparing attentional and inhibitory processing in adults with ADHD and adults with BD. In addition, since the majority of previous ERP studies on ADHD have used male samples (McLoughlin *et al.* 2010, 2011; Albrecht *et al.* 2013; Doehnert *et al.* 2013), and very few studies of this kind have been conducted in BD, our all-female sample furthers our understanding of neurophysiological impairments in females with either of these disorders.

Our ERP results show a significant difference between the ADHD and BD groups in the amplitude of the N2 in response to NoGo stimuli, which was reduced in participants with BD compared with the other two groups. The N2 is considered to reflect conflict-monitoring processing (Holroyd *et al.* 2003; Yeung & Cohen, 2006) and to depend on the amount of correct response processing needed to overcome a conflicting response. In the CPT-OX, this process may be represented by the bias towards the response after a cue, which requires the preparation of a response, and produces increased conflict monitoring when the prepared response has to be stopped in presence of a non-target. The reduced N2 in women with BD aligns with previous evidence of attenuated N2 elicited with an oddball task (Ethridge *et al.* 2012) and of a reduced ERN in error responses (Morsel *et al.* 2014). Both N2 and ERN in conditions inducing conflict, such as in non-target or incongruent trials, are thought to reflect conflict monitoring (Yeung & Cohen, 2006). Our results may therefore indicate that women with BD show impaired conflict monitoring compared with women with ADHD and control women. In line with previous studies using the CPT-OX (McLoughlin *et al.* 2010, 2011; Albrecht *et al.* 2013; Doehnert *et al.* 2013), we did not find an attenuated NoGo-N2 in women with ADHD, although reduced N2 have been associated with ADHD in tasks inducing higher conflict demands (McLoughlin *et al.* 2009, 2014*b*).

We also identified abnormalities in ERPs that distinguished women in both clinical groups from controls, indicating shared neurophysiological impairments across ADHD and BD. The reduced P3 in response to NoGo stimuli in both ADHD and BD groups, compared with the control group, suggests a similar pattern of impaired response inhibition to that previously reported in investigations of children and adults with ADHD (McLoughlin *et al.* 2010, 2011; Albrecht *et al.* 2013; Doehnert *et al.* 2013). The reduced NoGo-P3 in women with BD also aligns with previous cognitive research finding deficits in inhibitory control in euthymic BD (Robinson *et al.* 2006, 2013). These attenuations of the NoGo-P3 in both disorders therefore probably represent an area of overlapping impairment in brain processes implicated in the inhibition of incorrect response. Yet, this inhibitory control deficit in women with BD was temporally preceded by other processing deficits in the NoGo-N2. As such, in ERPs to non-targets, while women with ADHD seem primarily impaired in response inhibition, women with BD show a broader deficit in both conflict monitoring and inhibitory control.

Additionally, we report an attenuation in the CNV in women with ADHD compared with controls, and also potentially in women with BD (trend-level difference), both with a medium effect size. These results replicate previous studies reporting reduced CNV in individuals with ADHD (McLoughlin *et al.* 2010, 2011; Albrecht *et al.* 2013; Doehnert *et al.* 2013; Tye *et al.* 2014), and suggest another potential area of shared impairment with BD. However, we note that the comparison between BD and control participants was only at trend level. If replicated also in BD, this attenuation of the CNV would index an overlapping impairment in response preparation in the two disorders.

The lack of a difference between women with ADHD and controls in the Cue-P3 is inconsistent with some previous investigations showing a reduced Cue-P3 in ADHD samples (McLoughlin *et al.* 2010; Albrecht *et al.* 2013). Yet, these attenuations have not been reported in all studies (Dhar *et al.* 2010; Skirrow, 2012) and the difference in the Cue-P3 emerged as significant, but with a small effect size, in a recent larger-scale study of adolescents and young adults with ADHD (Cheung *et al.* 2015). In the present study, the normal Cue-P3 in ADHD may be due to an effect of gender, the current study being the first using an all-female sample. An age-effect is also plausible, since this study included adults of a slightly older and broader age range compared with previous investigations (e.g. McLoughlin *et al.* 2010) and developmental changes have been reported for the Cue-P3, suggesting that ADHD–control differences may decline with age (Doehnert *et al.* 2013). Further studies on larger samples that include participants of both genders and a broader age range are needed to clarify potential gender and age effects on these processes in ADHD.

While ERP measures of conflict monitoring differentiated the ADHD and BD groups, cognitive performance data did not suggest differences between the two clinical groups. Our cognitive performance results potentially suggest poorer performance and higher RTV in both ADHD and BD groups, compared with controls, consistent with previous studies reporting lower accuracy and higher RTV in ADHD and BD independently (Brotman *et al.* 2009; Kuntsi *et al.* 2010; Torralva *et al.* 2011). This pattern of results, with differences between ADHD and BD groups observed in the neurophysiological markers but not at the cognitive performance level, may reflect greater specificity of the neurophysiological markers in detecting differences between clinical groups.

The following limitations of this study should be taken into account when interpreting these data. First, although the groups were matched on gender, age and IQ, there were differences in the prescribed medications that participants with ADHD or BD were taking. While we asked participants with ADHD to stop taking stimulant medications 48 h prior to the assessment, it was not possible, for ethical reasons, to ask participants to stop mood-stabilizing, anti-psychotic or antidepressant medications. Given limited numbers in medication subgroups, we were not able to directly test the effect of medication on ERP measures, which represents a limitation of the current study. The effects of medication are difficult to control for in cross-disorder comparison studies where different treatments may be prescribed to different groups of psychiatric patients. Although the understanding of the effects of medications on ERPs is still limited, previous studies suggest that medications may normalize ERP measures (Anderer *et al.* 2002; Karaaslan *et al.* 2003; Galletly *et al.* 2005). As such, in this study, a medication effect could potentially have resulted in ERPs comparable with controls. Yet, both clinical groups, although some participants were medicated, showed reduced ERP measures compared with controls. Therefore, although the effect of medication represents a potential confounder of this study and may have attenuated some case–control differences, we report impairments in both clinical groups which may not have been produced by the effect of medication. Future studies on samples including non-medicated individuals or a higher number of individuals in each medication subgroup are needed to clarify whether our results may have been affected by medication effects. A second limitation is that, by using an area measure, we were not able to obtain latency data. This approach, previously adopted in similar ERP studies (Groom *et al.* 2010; Tye *et al.* 2014), was preferred for having the advantage, over peak measures, of being unaffected by latency variability and of providing a reliable measure of amplitude even when the identification of clear peaks is not possible for all subjects (Luck, 2005). Although some previous studies found prolonged latency of ERP components in BD (Chun *et al.* 2013; Maekawa *et al.* 2013), our ERP grand averages did not suggest latency differences, thus our area measure probably captured most of the differences between the groups on ERP measures. Finally, in order to increase homogeneity of the sample, this investigation was conducted on an all-female sample, with slightly higher than expected IQ in the clinical groups. Replication in future investigations with bigger samples of both genders and including individuals with a wider range of IQs is required in order to generalize these findings to more typical clinical populations.

## Conclusion

In conclusion, our results represent some of the first evidence of disorder-specific and shared impairments in brain processes involved in attentional orienting, conflict monitoring and inhibitory control in women with ADHD and BD, with moderate to large effect sizes. This investigation of neurophysiological processes furthers our understanding of impairments associated with ADHD and BD, and the identification of objective measures showing differences between ADHD and BD may assist in differentiating between the two disorders when their distinction is not clear at clinical consultations. If replicated in larger-scale studies, the neurophysiological biomarkers of distinct patterns in brain activity may aid in the identification of the diagnostic boundaries of ADHD and BD in adults. More broadly, given that ADHD and BD are both highly heritable disorders, the identified neurophysiological indices may represent intermediate phenotypes between diagnosis and genetic factors influencing a disorder, as suggested by genetic and family studies on ERP indices of attentional and inhibitory processing showing shared familial/genetic influences with ADHD (McLoughlin *et al.* 2011; Albrecht *et al.* 2013, 2014). Future studies can investigate causal models of ADHD and BD, by exploring to what extent overlapping and disorder-specific impairments in brain function are accounted for by specific or shared genetic influences on the two disorders and, in turn, further our understanding on the pathways to distinct and overlapping features in ADHD and BD.

## Note

1 Since most previous ERP analyses on CPT-OX did not apply a baseline subtraction (Banaschewski *et al.* 2004; McLoughlin *et al.* 2010, 2011; Albrecht *et al.* 2013; Doehnert *et al.* 2013), analyses were also repeated without baseline correction. Results of data without baseline correction were comparable for the NoGo-N2, NoGo-P3 and Go-P3, but partly changed for the Cue-P3 and CNV (see Supplementary material).

## Footnotes

† The notes appear after the main text.

## Supplementary material

For supplementary material accompanying this paper visit http://dx.doi.org/10.1017/S0033291715001877. click here to view supplementary material
