# Supplementary material for: Disorder-specific and shared neurophysiological impairments of attention and inhibition in women with attention-deficit/hyperactivity disorder and women with bipolar disorder
Source: Psychol Med. 2015 Nov 10;46(3):493–504. doi: 10.1017/S0033291715001877 (PMC4697305; doi:10.1017/S0033291715001877)
Supplement: Supplementary file 1 [file S0033291715001877sup001.docx]

**SUPPLEMENTARY MATERIAL**

**Number of artefact-free segments included in each condition**

The average number of segments in each group for the Cue, NoGo and Go conditions is reported in Supplementary table 1. The number of segments was entered into univariate ANOVA to check for group differences, with ‘group’ as between-subjects variable (ADHD, BD and control participants). Groups did not differ on the number of artefact-free segments for the Cue condition [F(2, 57)=0.30, p=0.75], the NoGo condition [F(2, 57)=0.15, p=0.87] or Go condition [F(2, 55)=0.49, p=0.62].

**Analysis of ERP parameters without baseline correction**

The majority of previous ERP analyses on CPT-OX in ADHD samples did not apply a baseline subtraction (Banaschewski *et al.* 2004; McLoughlin *et al.* 2010, 2011; Albrecht *et al.* 2013; Doehnert *et al.* 2013). In this study, we chose to apply a baseline correction in order to reduce the influence of pre-stimulus activity on our ERP measures. However, analyses were also repeated without baseline correction in order to allow comparison with previous results.

### *Cue condition*

A trend-level effect of group emerged for the Cue-P3 [F(2,57)=2.48, p=0.09]. Post-hoc comparisons showed a significant difference between the ADHD and the BD group (p=0.03), with large effect size (Supplementary table 2). The control group did not differ from either the ADHD (p=0.59) or the BD (0.14) groups.

A significant effect of group emerged for the CNV [F(2,57)=3.68, p=0.03]. Post-hoc comparisons showed a significant difference between the ADHD and the control group (p=0.01), with large effect size (Supplementary table 2). The BD group did not differ from either the ADHD (p=0.22) or the control (0.15) groups.

###

### *NoGo condition*

A significant effect of group on the NoGo-N2 [F(2,57)=5.12, p=0.01]. Post-hoc analyses revealed that BD participants significantly differed from ADHD (p=0.01) and control (p=0.02) participants, both with large effect sizes (Supplementary table 2). The ADHD and the control groups did not differ from each other (p=0.68).

A significant effect of group emerged on the NoGo-P3 [F(2,57)=3.35, p=0.04]. Post-hoc analyses showed that both ADHD (p=0.05) and BD (p=0.02) participants significantly differed from controls, respectively with medium and large effect sizes (Supplementary table 2), but not from each other (p=0.55).

### *Go condition*

No significant effect of group emerged on the Go-P3 [F(2,55)=0.61, p=0.55].

**Comparison with results of data with baseline correction**

Results of data without baseline correction (Supplementary table 2) showed a reduced Cue-P3 in participants with BD compared to participants with ADHD, which was not observed in data with baseline correction. No difference emerged between the BD and control groups in the CNV, which was at trend level in results of data with baseline correction. Group differences in ERPs from the NoGo and Go conditions remained the same.

Of note, an ADHD-control difference in the Cue-P3 was not found when analysing data with or without baseline correction. Although this difference has been reported in previous studies using this task when a baseline subtraction was not applied (Banaschewski *et al.* 2004; McLoughlin *et al.* 2010, 2011; Albrecht *et al.* 2013; Doehnert *et al.* 2013), this discrepancy is likely not due to the use of baseline correction. Possible explanations for the lack of ADHD-control difference in the Cue-P3 in this sample are discussed in the main text (see Discussion section).

**SUPPLEMENTARY TABLES**

**Supplementary table 1** Mean (SD) number of artefact-free segments in each ERP average by group and condition during the CPT-OX.

|  | **ADHD (n=20)***  mean (SD) | **BD (n=20)**  mean (SD) | **Controls (n=20)**  mean (SD) |
| --- | --- | --- | --- |
| **Cue** | 58.35 (11.65) | 60.10 (10.28) | 60.80 (9.05) |
| **NoGo** | 30.75 (3.78) | 30.05 (4.73) | 30.30 (3.85) |
| **Go** | 29.22 (5.99) | 29.20 (5.29) | 30.65 (4.87) |

Abbreviations: ADHD, attention deficit hyperactivity disorder; BD, bipolar disorder.

*Only 18 ADHD participants were included in the average of the Go condition, as two subjects did not have at least 20 artefact-free segments.

**Supplementary table 2** ERP measures from the CPT-OX (without baseline correction): means (SDs), effect sizes (Cohen’s d) and significance of group comparisons.

|  | **ADHD (n=20)***  mean (SD) | **BD (n=20)**  mean (SD) | **Controls (n=20)**  mean (SD) | **ADHD vs. BD**  effect size (d) | **ADHD vs. Controls** effect size (d) | **BD vs. Controls** effect size (d) |
| --- | --- | --- | --- | --- | --- | --- |
| **Cue-P3 at Pz** | 1.73 (1.37) | 0.72 (1.41) | 1.47 (1.67) | **0.75*** | 0.18 | *0.50* |
| **CNV at Cz** | -2.33 (1.02) | -2.79 (1.29) | -3.50 (1.74) | 0.41 | **0.85*** | *0.48* |
| **NoGo-N2 at Fz** | -0.45 (0.96) | 0.90 (1.94) | -0.64 (1.88) | **0.90*** | 0.13 | **0.83*** |
| **NoGo-P3 at Cz** | 3.33 (1.92) | 2.93 (2.34) | 4.50 (1.69) | 0.19 | *0.67** | **0.79*** |
| **Go-P3 at CPz** | 2.77 (2.76) | 3.20 (2.85) | 3.63 (2.11) | 0.17 | 0.41 | 0.18 |

Note: mean and SD were calculated on raw data. Large effect sizes are given in bold, medium effect sizes are given in italics; *p<0.05, †p<0.10; results changing compared to analysis of data with baseline correction are underlined.

Abbreviations: ADHD, attention deficit hyperactivity disorder; BD, bipolar disorder; CNV, contingent negative variation.

*Only 18 participants with ADHD were included in the average of the Go condition, as two participants did not have at least 20 artefact-free segments.
